# (*Z*)-4,5-Di­bromo-3,3,6,6-tetra­methyl-2,3,6,7-tetra­hydro­thiepine-1,1-dione

**DOI:** 10.1107/S2414314623000421

**Published:** 2023-01-31

**Authors:** Dieter Schollmeyer, Heiner Detert

**Affiliations:** a University of Mainz, Department of Chemistry, Duesbergweg 10-14, 55099 Mainz, Germany; Goethe-Universität Frankfurt, Germany

**Keywords:** crystal structure, strain, bromine, heterocycles

## Abstract

The crystal of the title compound, C_10_H_16_Br_2_O_2_S, is formed from layers built from centrosymmetric pairs of mol­ecules. The mol­ecule adopts a twist conformation with the carbon atoms next to sulfur above or below the mean plane.

## Abstract 2

The crystal of the title compound is formed from layers built from centrosymmetric pairs of mol­ecules. The mol­ecule adopts a twist conformation with the carbons next to sulfur above or below the mean plane.

## Graphical abstract

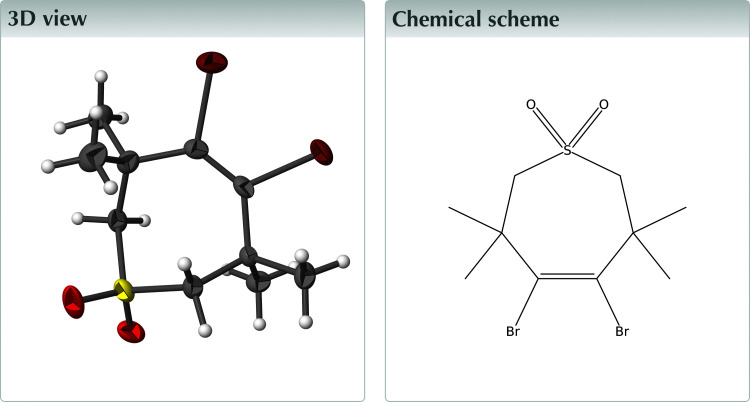

## Structure description

**Figure 1:**
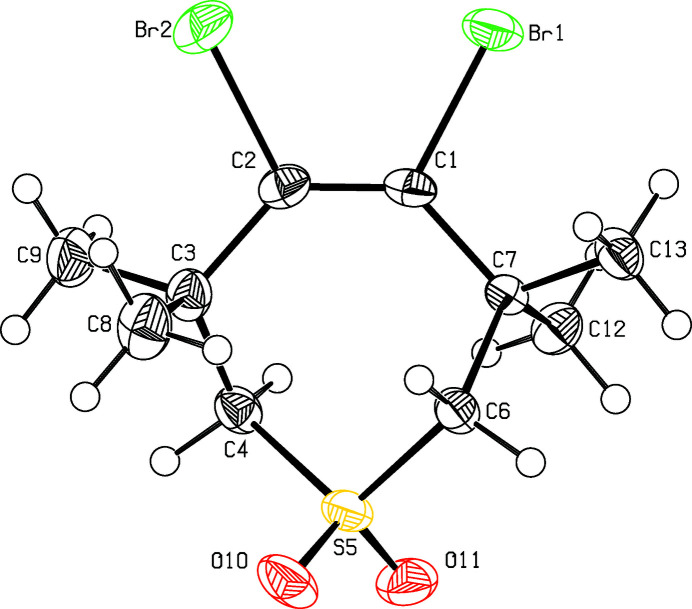
View of the title compound. Displacement ellipsoids are drawn at the 50% probability level.

**Figure 2:**
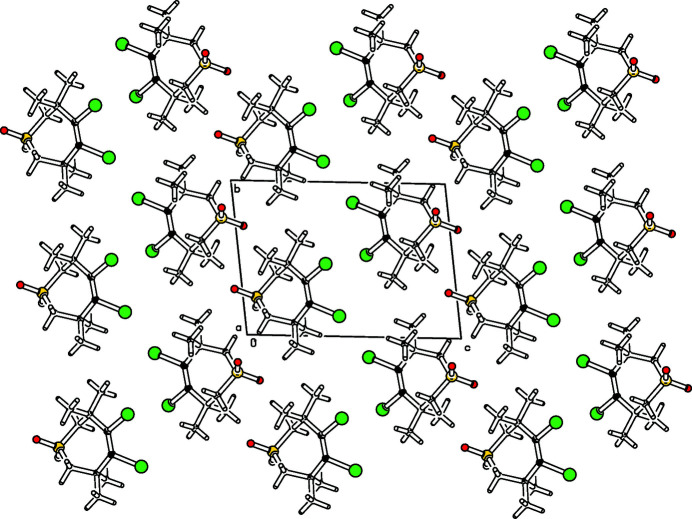
Partial packing diagram. View along the *a*-axis.

As part of our studies on the reactivity of angle-strained compounds (Krämer *et al.*, 2009 ▸; Detert 2011 ▸), the addition of bromine appeared to be a challenging project (Chiappe *et al.*, 2002 ▸; Detert *et al.* 1992 ▸). Whereas the addition of bromine to alkynes generally leads *via* bridged bromo­nium ions to *trans*-di­bromo­alkenes, the bromination of cyclo­octyne gives *cis*-1,2-di­bromo­cyclo­octene (Wittig & Dorsch, 1968 ▸). While this can proceed *via* isomerization of the initially formed *trans* isomer, the addition of bromine to cyclo­heptynes avoids cationic inter­mediates (Herges *et al.* 2005 ▸). The title compound (Fig. 1 ▸) was obtained within these studies *via* addition of bromine to tetra­methyl­thia­cyclo­heptyne-*S,S*-dioxide (Krebs *et al.* 1979 ▸). Two identical, non-symmetrical mol­ecules comprise the unit cell. The conformation of the seven-membered ring is similar to a twist form. The atoms C7,C1,C3,S5 are nearly coplanar with the largest deviation from planarity at C1 [0.056 (3) Å]. The atoms vicinal to sulfur adopt positions below [−0.789 (3) Å, C4] and above [0.785 (3) Å, C6] this plane. The tetra­substituted olefin is twisted, torsion angle C7—C1—C2—C3 is −13.7 (6)° and Br1—C1—C2—Br2 at −15.3 (3)° is even larger. Two mol­ecules are connected by a center of inversion, the packing appears as a layer structure (Fig. 2 ▸). Layers are parallel to the *a* axis, the minimal distance between bromine atoms (Br1⋯Br1′) of different layers is 3.4168 (6) Å.

## Synthesis and crystallization

The title compound C_10_H_16_O_2_Br_2_S was prepared from the cyclic alkyne (Krebs *et al.*, 1979 ▸; Krebs & Colberg 1980 ▸) by addition of bromine at 203 K according to the procedure given by Herges *et al.* (2005 ▸). After evaporation of the solvent, the oily compound crystallized after standing for 15 years at ambient temperature.

## Refinement

Crystal data, data collection and structure refinement details are summarized in Table 1 ▸.

## Supplementary Material

Crystal structure: contains datablock(s) I, global. DOI: 10.1107/S2414314623000421/bt4132sup1.cif

Structure factors: contains datablock(s) I. DOI: 10.1107/S2414314623000421/bt4132Isup2.hkl

Supporting information file. DOI: 10.1107/S2414314623000421/bt4132Isup3.cml

CCDC reference: 2236694

Additional supporting information:  crystallographic information; 3D view; checkCIF report

## Figures and Tables

**Table 1:** Experimental details

Crystal data	
Chemical formula	C_10_H_16_Br_2_O_2_S
*M*_r_	360.11
Crystal system, space group	Triclinic, *P* 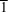
Temperature (K)	193
*a*, *b*, *c* (Å)	5.9685 (4), 8.9642 (6), 12.4927 (9)
α, β, γ (°)	94.804 (6), 102.448 (5), 99.356 (5)
*V* (Å^3^)	639.09 (8)
*Z*	2
Radiation type	Mo *K*α
μ (mm^−1^)	6.49
Crystal size (mm)	0.67 × 0.39 × 0.08

Data collection	
Diffractometer	Stoe *IPDS* 2T
Absorption correction	Integration (*X-RED*; Stoe et al., 2019 ▸)
*T*_min_, *T*_max_	0.088, 0.553
No. of measured, independent and observed [*I* > 2σ(*I*)] reflections	8129, 3038, 2686
*R*_int_	0.023
(sin θ/λ)_max_ (Å^−1^)	0.663

Refinement	
*R*[*F* ^2^ > 2σ(*F* ^2^)], *wR*(*F* ^2^), *S*	0.036, 0.089, 1.14
No. of reflections	3038
No. of parameters	140
H-atom treatment	H-atom parameters constrained
Δρ_max_, Δρ_min_ (e Å^−3^)	1.28, −0.89

Computer programs: *X-AREA* *WinXpose*, *Recipe* and *Integrate* (Stoe & Cie, 2019 ▸), *SHELXT2014* (Sheldrick, 2015*a* ▸), *SHELXL2018/3* (Sheldrick, 2015*b* ▸) and *PLATON* (Spek, 2020 ▸).

## full crystallographic data

### Crystal data

**Table:**

C_10_H_16_Br_2_O_2_S	*Z* = 2
*M**_r_* = 360.11	*F*(000) = 356
Triclinic, *P*1	*D*_x_ = 1.871 Mg m^−^^3^
*a* = 5.9685 (4) Å	Mo *K*α radiation, λ = 0.71073 Å
*b* = 8.9642 (6) Å	Cell parameters from 18448 reflections
*c* = 12.4927 (9) Å	θ = 2.7–28.4°
α = 94.804 (6)°	µ = 6.49 mm^−^^1^
β = 102.448 (5)°	*T* = 193 K
γ = 99.356 (5)°	Plate, colourless
*V* = 639.09 (8) Å^3^	0.67 × 0.39 × 0.08 mm

### Data collection

**Table:**

Stoe IPDS 2T diffractometer	3038 independent reflections
Radiation source: sealed X-ray tube, 12 x 0.4 mm long-fine focus	2686 reflections with *I* > 2σ(*I*)
Detector resolution: 6.67 pixels mm^-1^	*R*_int_ = 0.023
rotation method, ω scans	θ_max_ = 28.1°, θ_min_ = 2.7°
Absorption correction: integration (XRED; Stoe et al., 2019)	*h* = −7→7
*T*_min_ = 0.088, *T*_max_ = 0.553	*k* = −11→11
8129 measured reflections	*l* = −16→16

### Refinement

**Table:**

Refinement on *F*^2^	Primary atom site location: dual
Least-squares matrix: full	Hydrogen site location: inferred from neighbouring sites
*R*[*F*^2^ > 2σ(*F*^2^)] = 0.036	H-atom parameters constrained
*wR*(*F*^2^) = 0.089	*w* = 1/[σ^2^(*F*_o_^2^) + (0.0307*P*)^2^ + 1.4492*P*] where *P* = (*F*_o_^2^ + 2*F*_c_^2^)/3
*S* = 1.14	(Δ/σ)_max_ < 0.001
3038 reflections	Δρ_max_ = 1.28 e Å^−^^3^
140 parameters	Δρ_min_ = −0.89 e Å^−^^3^
0 restraints	

### Special details

**Table:**

Geometry. All esds (except the esd in the dihedral angle between two l.s. planes) are estimated using the full covariance matrix. The cell esds are taken into account individually in the estimation of esds in distances, angles and torsion angles; correlations between esds in cell parameters are only used when they are defined by crystal symmetry. An approximate (isotropic) treatment of cell esds is used for estimating esds involving l.s. planes.
Refinement. Hydrogen atoms attached to carbons were placed at calculated positions and were refined in the riding-model approximation with C–H = 0.95 Å, and with *U*_iso_(H) = 1.2 *U*_eq_(C).

### Fractional atomic coordinates and isotropic or equivalent isotropic displacement parameters (Å^2^)

**Table:**

	*x*	*y*	*z*	*U*_iso_*/*U*_eq_	
Br1	0.15368 (7)	0.46873 (4)	0.40230 (3)	0.03826 (12)	
Br2	0.21628 (8)	0.13145 (5)	0.44057 (3)	0.04584 (13)	
C1	0.2846 (5)	0.3563 (4)	0.3004 (2)	0.0251 (6)	
C2	0.2791 (5)	0.2074 (4)	0.3085 (3)	0.0265 (6)	
C3	0.3191 (6)	0.0802 (4)	0.2281 (3)	0.0281 (7)	
C4	0.2643 (5)	0.1158 (4)	0.1074 (3)	0.0266 (6)	
H4A	0.115877	0.154728	0.094409	0.032*	
H4B	0.235588	0.018273	0.058883	0.032*	
S5	0.46965 (15)	0.24522 (9)	0.06328 (7)	0.02945 (18)	
C6	0.5605 (5)	0.3987 (3)	0.1695 (3)	0.0254 (6)	
H6A	0.674923	0.368072	0.229620	0.031*	
H6B	0.645465	0.484410	0.140356	0.031*	
C7	0.3767 (5)	0.4610 (3)	0.2221 (3)	0.0226 (6)	
C8	0.5636 (6)	0.0428 (5)	0.2667 (4)	0.0401 (9)	
H8A	0.586667	−0.036909	0.213729	0.060*	
H8B	0.578609	0.006994	0.339500	0.060*	
H8C	0.681643	0.134537	0.271694	0.060*	
C9	0.1385 (7)	−0.0676 (4)	0.2226 (4)	0.0396 (8)	
H9A	0.155064	−0.144330	0.165629	0.059*	
H9B	−0.019675	−0.045063	0.204374	0.059*	
H9C	0.165983	−0.106587	0.294348	0.059*	
O10	0.6724 (5)	0.1800 (3)	0.0569 (3)	0.0434 (7)	
O11	0.3486 (5)	0.2945 (3)	−0.0361 (2)	0.0416 (6)	
C12	0.1749 (6)	0.4981 (4)	0.1357 (3)	0.0300 (7)	
H12A	0.238109	0.561693	0.085059	0.045*	
H12B	0.078319	0.553061	0.172897	0.045*	
H12C	0.079307	0.403256	0.093870	0.045*	
C13	0.5228 (6)	0.6125 (4)	0.2895 (3)	0.0315 (7)	
H13A	0.593338	0.675014	0.240333	0.047*	
H13B	0.646160	0.589899	0.348092	0.047*	
H13C	0.420881	0.668146	0.322457	0.047*	

### Atomic displacement parameters (Å^2^)

**Table:**

	*U*^11^	*U*^22^	*U*^33^	*U*^12^	*U*^13^	*U*^23^
Br1	0.0480 (2)	0.0446 (2)	0.03061 (19)	0.01935 (16)	0.02005 (15)	0.00268 (15)
Br2	0.0666 (3)	0.0435 (2)	0.0296 (2)	0.00595 (19)	0.01546 (18)	0.01398 (16)
C1	0.0262 (14)	0.0332 (16)	0.0169 (13)	0.0094 (12)	0.0058 (11)	0.0003 (12)
C2	0.0254 (15)	0.0331 (16)	0.0230 (15)	0.0073 (12)	0.0064 (12)	0.0086 (13)
C3	0.0255 (15)	0.0251 (15)	0.0356 (18)	0.0066 (12)	0.0088 (13)	0.0062 (13)
C4	0.0242 (14)	0.0244 (15)	0.0296 (16)	0.0013 (12)	0.0075 (12)	−0.0034 (12)
S5	0.0316 (4)	0.0281 (4)	0.0289 (4)	−0.0004 (3)	0.0151 (3)	−0.0049 (3)
C6	0.0250 (14)	0.0226 (14)	0.0285 (16)	0.0033 (11)	0.0083 (12)	−0.0011 (12)
C7	0.0267 (14)	0.0217 (14)	0.0209 (14)	0.0066 (11)	0.0079 (11)	0.0008 (11)
C8	0.0314 (18)	0.0378 (19)	0.055 (2)	0.0150 (15)	0.0094 (16)	0.0136 (17)
C9	0.041 (2)	0.0278 (17)	0.052 (2)	0.0009 (15)	0.0191 (17)	0.0054 (16)
O10	0.0377 (14)	0.0372 (14)	0.0587 (18)	0.0019 (11)	0.0290 (13)	−0.0124 (12)
O11	0.0546 (16)	0.0428 (15)	0.0247 (12)	−0.0024 (12)	0.0133 (11)	0.0007 (11)
C12	0.0280 (16)	0.0327 (17)	0.0300 (17)	0.0070 (13)	0.0052 (13)	0.0087 (13)
C13	0.0373 (18)	0.0261 (16)	0.0291 (17)	0.0066 (13)	0.0053 (14)	−0.0034 (13)

### Geometric parameters (Å, º)

**Table:**

Br1—C1	1.927 (3)	C6—H6B	0.9900
Br2—C2	1.923 (3)	C7—C12	1.536 (4)
C1—C2	1.343 (5)	C7—C13	1.556 (4)
C1—C7	1.536 (4)	C8—H8A	0.9800
C2—C3	1.538 (5)	C8—H8B	0.9800
C3—C8	1.535 (5)	C8—H8C	0.9800
C3—C4	1.543 (5)	C9—H9A	0.9800
C3—C9	1.552 (5)	C9—H9B	0.9800
C4—S5	1.758 (3)	C9—H9C	0.9800
C4—H4A	0.9900	C12—H12A	0.9800
C4—H4B	0.9900	C12—H12B	0.9800
S5—O11	1.438 (3)	C12—H12C	0.9800
S5—O10	1.441 (3)	C13—H13A	0.9800
S5—C6	1.758 (3)	C13—H13B	0.9800
C6—C7	1.547 (4)	C13—H13C	0.9800
C6—H6A	0.9900		
			
C2—C1—C7	132.0 (3)	C1—C7—C12	111.0 (3)
C2—C1—Br1	117.5 (2)	C1—C7—C6	112.8 (2)
C7—C1—Br1	110.4 (2)	C12—C7—C6	112.6 (3)
C1—C2—C3	130.6 (3)	C1—C7—C13	109.7 (3)
C1—C2—Br2	118.0 (2)	C12—C7—C13	108.7 (3)
C3—C2—Br2	111.4 (2)	C6—C7—C13	101.5 (2)
C8—C3—C2	110.8 (3)	C3—C8—H8A	109.5
C8—C3—C4	113.7 (3)	C3—C8—H8B	109.5
C2—C3—C4	112.0 (3)	H8A—C8—H8B	109.5
C8—C3—C9	107.6 (3)	C3—C8—H8C	109.5
C2—C3—C9	110.2 (3)	H8A—C8—H8C	109.5
C4—C3—C9	102.1 (3)	H8B—C8—H8C	109.5
C3—C4—S5	119.1 (2)	C3—C9—H9A	109.5
C3—C4—H4A	107.5	C3—C9—H9B	109.5
S5—C4—H4A	107.5	H9A—C9—H9B	109.5
C3—C4—H4B	107.5	C3—C9—H9C	109.5
S5—C4—H4B	107.5	H9A—C9—H9C	109.5
H4A—C4—H4B	107.0	H9B—C9—H9C	109.5
O11—S5—O10	116.84 (18)	C7—C12—H12A	109.5
O11—S5—C4	107.11 (16)	C7—C12—H12B	109.5
O10—S5—C4	110.42 (17)	H12A—C12—H12B	109.5
O11—S5—C6	109.96 (16)	C7—C12—H12C	109.5
O10—S5—C6	106.90 (16)	H12A—C12—H12C	109.5
C4—S5—C6	105.01 (15)	H12B—C12—H12C	109.5
C7—C6—S5	119.5 (2)	C7—C13—H13A	109.5
C7—C6—H6A	107.4	C7—C13—H13B	109.5
S5—C6—H6A	107.4	H13A—C13—H13B	109.5
C7—C6—H6B	107.4	C7—C13—H13C	109.5
S5—C6—H6B	107.4	H13A—C13—H13C	109.5
H6A—C6—H6B	107.0	H13B—C13—H13C	109.5
			
C7—C1—C2—C3	−13.7 (6)	C3—C4—S5—O10	70.2 (3)
Br1—C1—C2—C3	165.7 (3)	C3—C4—S5—C6	−44.7 (3)
C7—C1—C2—Br2	165.3 (3)	O11—S5—C6—C7	71.3 (3)
Br1—C1—C2—Br2	−15.3 (3)	O10—S5—C6—C7	−161.0 (3)
C1—C2—C3—C8	102.4 (4)	C4—S5—C6—C7	−43.7 (3)
Br2—C2—C3—C8	−76.6 (3)	C2—C1—C7—C12	105.5 (4)
C1—C2—C3—C4	−25.7 (5)	Br1—C1—C7—C12	−73.9 (3)
Br2—C2—C3—C4	155.3 (2)	C2—C1—C7—C6	−21.9 (5)
C1—C2—C3—C9	−138.6 (4)	Br1—C1—C7—C6	158.6 (2)
Br2—C2—C3—C9	42.4 (3)	C2—C1—C7—C13	−134.3 (4)
C8—C3—C4—S5	−48.3 (4)	Br1—C1—C7—C13	46.3 (3)
C2—C3—C4—S5	78.3 (3)	S5—C6—C7—C1	74.8 (3)
C9—C3—C4—S5	−163.9 (2)	S5—C6—C7—C12	−51.8 (3)
C3—C4—S5—O11	−161.6 (2)	S5—C6—C7—C13	−167.8 (2)
